# Interleukin-31 as a Clinical Target for Pruritus Treatment

**DOI:** 10.3389/fmed.2021.638325

**Published:** 2021-02-12

**Authors:** Kenji Kabashima, Hiroyuki Irie

**Affiliations:** Department of Dermatology, Graduate School of Medicine, Kyoto University, Kyoto, Japan

**Keywords:** interleukin-31, interleukin-31 receptor, pruritus, itch, anti-IL-31, anti-IL-31RA, nemolizumab

## Abstract

In recent years, the published literature has suggested the key involvement of the cytokine interleukin-31 (IL-31) in the symptomatology of pruritus, and both IL-31 and its receptor have become potential therapeutic targets for a range of pruritic diseases. Elevated levels of IL-31 or its receptor have been reported in the tissue or serum of patients with pruritic skin diseases, such as atopic dermatitis, prurigo nodularis, and psoriasis. Pruritus places a heavy burden on patients, and can have a negative impact on daily life, sleep, and mental health. Since current anti-pruritic treatments are often ineffective, affected patients are in urgent need of new therapies. As a result, drug development targeting the IL-31 pathway is evolving rapidly. To date, only nemolizumab, a humanized monoclonal antibody targeting the IL-31 receptor, has successfully completed late-stage clinical studies. This article will highlight our current clinical understanding of the role of IL-31 in pruritic disease, and explore recent progress in drug development as well as the anticipated future advances in this field.

## Introduction

The T-cell-derived cytokine interleukin-31 (IL-31) was first identified in 2004 (1). Investigation in animal models suggested a role for IL-31 in the cutaneous and epithelial signs and symptoms observed in pruritus, skin inflammation, and airway hypersensitivity (1). Overexpression of IL-31 has been shown to be associated with promotion of sensory neuronal outgrowth (2) and stimulation (3), providing increased sensitivity to minimal itch-inducing stimuli which can result in sustained pruritus. Expression of IL-31 in skin is also associated with a profound repression of the filaggrin protein, which is critical in the differentiation of keratinocytes and skin barrier maintenance (4).

**Figure 1:**
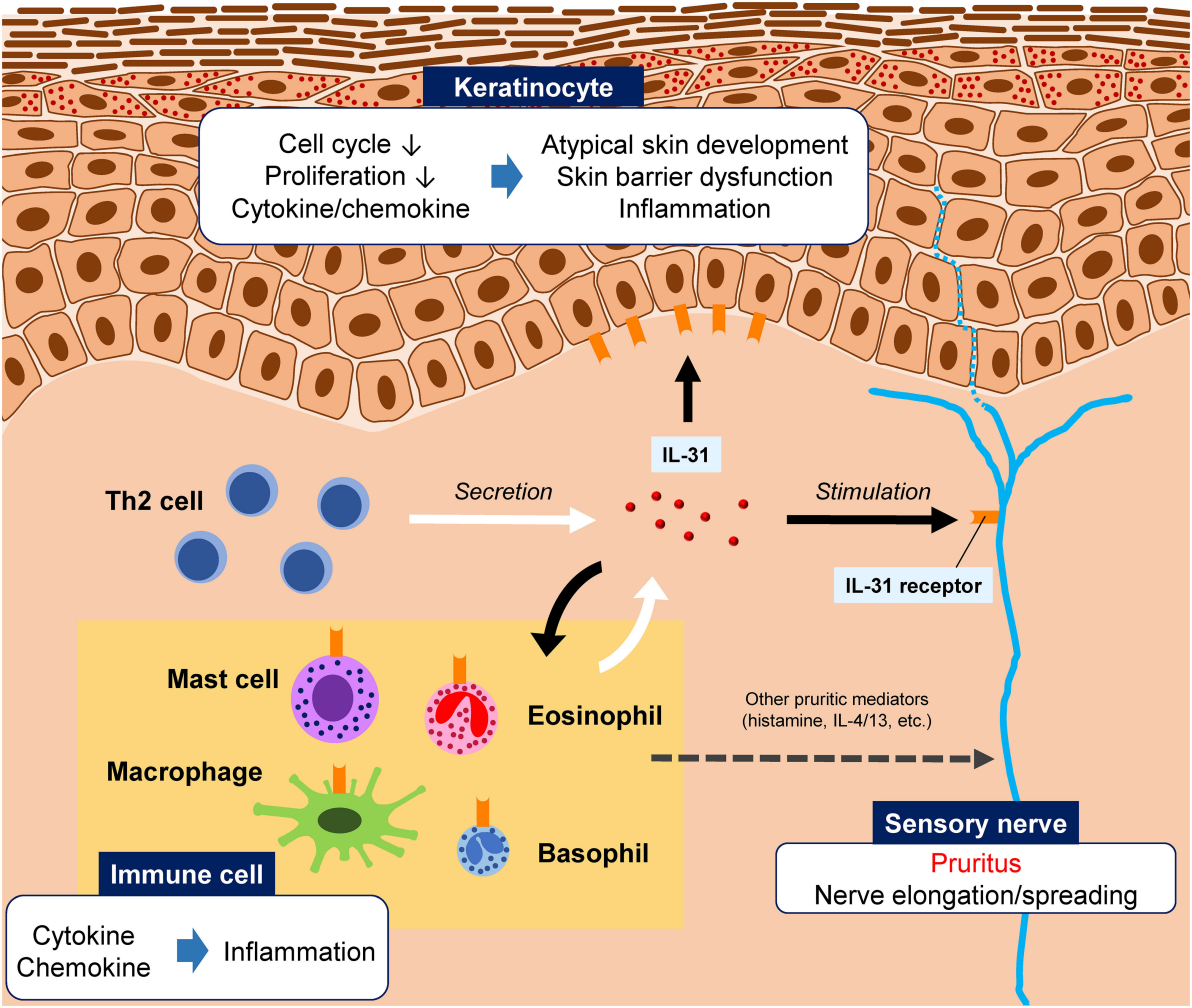
Illustration of the hypothesized roles of interleukin (IL)-31 in pruritic skin diseases. IL-31 is primarily produced by T helper 2 (Th2) cells, although other innate immune cells can also produce IL-31. The IL-31 receptor is widely expressed by various cell types, including peripheral sensory nerves, epidermal keratinocytes, and immune cells. IL-31 binding to its receptor on sensory neurons stimulates the nerve, causing pruritus. IL-31 is also involved skin barrier dysfunction and inflammation.

The receptor for IL-31 is a heterodimeric complex composed of IL-31 receptor A (IL-31RA) and the oncostatin M receptor (OSMR) (1, 5); the binding of IL-31 to its receptor activates cell signaling pathways including Jak/STAT, PI3K/AKT, and MAPK (5). In keratinocytes, IL-31 has been shown to induce cell cycle arrest, resulting in reduced proliferation, which in turn leads to atypical skin development, defects, and barrier dysfunction (4) (Figure 1). In other organs and physiologic systems, IL-31 has been linked to hematopoiesis and regulation of the immune response (5).

The recent published literature from both preclinical and clinical studies has suggested the key involvement of IL-31 in the symptomatology of acute and chronic pruritus (6–9). The objectives of this article are to highlight our current clinical understanding of the role of IL-31 in pruritic diseases, and explore recent progress in drug development and the anticipated future advances in this field. Itching is a symptom that greatly impairs quality of life, and is often not well-controlled with current treatments (10, 11). In addition to the induction of itch, the IL-31 pathway also stimulates the release of inflammatory mediators and reduces the expression of molecules involved in the skin barrier, resulting in additional skin signs and symptoms (4, 12–14). As a result, both IL-31 and its receptor have become potential therapeutic targets for a range of pruritic diseases (14–18).

## The Burden of Pruritus

Itch is the most common complaint among patients who attend dermatology clinics, and for many patients, pruritic symptoms are intractable despite medication (19). In one study, 90% of patients with chronic skin diseases reported that they had experienced pruritus, and that the intensity of itch was related to impairment in several areas of daily life, including sleep quality, work productivity, and mental health (20).

### Patient Burden

Chronic pruritus has been shown to be as debilitating as chronic pain in terms of its impact on quality of life, with more severe itch having a more profound negative effect on daily life (21). In a recent study of 132 North American adults with chronic pruritus, health performance was found to be significantly reduced compared with normative controls (*p* < 0.001) (22). In a cross-sectional study of 602 US adults with atopic dermatitis, the most burdensome symptom was reported to be itch (54.4%), and severe itch scores (measured using the patient-oriented scoring atopic dermatitis-itch scale) were associated with poor mental health scores (23). Suicidal ideation has been reported to be highly prevalent in patients with chronic pruritus (20), and several studies have shown that patients with pruritus are more likely to have anxiety and depression compared with non-pruritic controls (20, 24, 25).

Pruritus also impairs sleep quality (20, 26–28), likely due to both the effects of scratching and underlying systemic inflammation (29). In one analysis, chronic pruritic dermatoses were found to be associated with increased nighttime awakenings (odds ratio 1.329–1.646) (30), while another study of patients with psoriasis found that pruritus significantly increased the difficulty of falling asleep (*p* = 0.031) (27). Thus, treatments for pruritus that can also improve sleep and quality of life are urgently needed.

### Economic Burden

A study in North American adults calculated that chronic pruritus was associated with a mean of −5.5 quality-adjusted life-years per patient, which translated into an individual lifetime economic burden of US$275, and a societal burden of almost US$90 billion (22). Another study calculated median annualized costs of chronic pruritus of US$1067 per patient (31). In addition to the costs of clinic consultations, referrals, laboratory tests, and prescription medication (32), the scratching behavior associated with pruritus may be associated with skin breakdown and increased infection, resulting in increased direct medical costs from attendance at emergency departments and hospitalization (33, 34). Indirect costs associated with pruritus include reduced work or school productivity (absenteeism and presenteeism), over-the-counter treatments, and the time required to apply topical agents or perform other self-treatment (31).

## IL-31 in Disease

In the past few years, an increasing number of researchers have postulated a link between IL-31 and the manifestations of various diseases, both dermatologic and non-dermatologic (5, 14, 17, 35). In addition to the development of itch (12), dysregulation of IL-31 and its receptor is thought to underlie alterations in the skin barrier (thickening or breakdown) (13), as well as auto-immune and inflammatory signs and symptoms (14). Individual diseases, and the putative role(s) of IL-31 in these disorders, are summarized below.

### Atopic Dermatitis

In preclinical studies, mice treated with IL-31 exhibited skin lesions and scratching behavior similar to those seen in atopic dermatitis (1, 18, 36), and treatments targeting IL-31 were found to reduce scratching (37, 38). In clinical studies, levels of serum IL-31 have been found to be elevated in patients with atopic dermatitis, compared with healthy individuals (39–41), and decreased after cyclosporin treatment (42); furthermore, IL-31 levels were shown to correlate with disease severity and pruritic symptoms (39–41).

IL-31 receptors are expressed constitutively on the surface of keratinocytes, eosinophils and small diameter neurons (43). Keratinocyte levels of IL-31RA expressed in atopic dermatitis lesions have been shown to be higher than those of normal skin in healthy subjects (44, 45), while no difference in levels of OSMR has been observed (44), emphasizing the greater importance of IL-31RA in the pathogenesis of the disease.

### Prurigo Nodularis

Prurigo nodularis, a chronic disease presenting as one or more hyperkeratotic papules which are intensely pruritic, results in a difficult to treat itch-scratch cycle, and causes sleep disturbances and psychiatric comorbidities in affected patients (46). An analysis of skin samples from patients with various chronic inflammatory skin diseases revealed that the highest levels of IL-31 were located in lesional skin from individuals with prurigo nodularis, with an elevation of IL-31 mRNA almost 50-fold higher than that of skin from healthy individuals (47).

### Psoriasis

Like atopic dermatitis, psoriasis is an inflammatory skin disease affecting millions of people worldwide, and can have a negative impact on the mental health and quality of life of patients (48). A role for IL-31 in the pathogenesis of psoriasis has been suggested (49), with elevated levels of serum IL-31 observed in psoriatic patients compared with controls (8). Patients with early-onset psoriasis have greater levels of Il-31 gene induction compared with healthy controls, and even compared with patients with atopic dermatitis (50). However, the data are inconsistent; other studies have failed to show elevated levels of IL-31 in psoriasis (51), or any association between IL-31 and pruritus intensity in psoriatic patients (52).

### Cutaneous T-Cell Lymphoma (CTCL)

CTCL comprises a heterogeneous group of diseases arising from the T cells involved in tumor responses (53). Most patients (around 88%) with CTCL are affected by pruritus (54), and studies have demonstrated elevated levels of IL-31, IL-31RA, and OSMRβ within the epidermis of affected patients (54), as well as increased serum IL-31 levels (55, 56). In one analysis, levels of IL-31 mRNA appeared to be correlated with the severity of pruritus in CTCL patients (57), although data from another study did not support this contention (58). It has been hypothesized that IL-31 may induce epidermal neoplastic T cells and keratinocytes to transmit itch, indirectly affecting sensory nerves (54). Although IL-31 appears to have a role in pruritus in CTCL, there is conflicting evidence as to whether it plays a larger role in the pathogenesis of CTCL itself (56, 58, 59). However, of note, chronic prurigo has also been reported in cases of Hodgkin lymphoma (HL) (60), and a study in HL patients demonstrated elevated IL-31 in HL cells and in the immune cells infiltrating affected lymph nodes (61). Another recent study showed that plasma concentrations of IL-31 decreased in HL patients entering remission (62); taken together, these findings suggest that IL-31 may contribute to immune suppression in HL, and may indicate a similar role in CTCL.

### Other Pruritic Disorders

#### Uremic Pruritus

Uremic pruritus occurs in patients with chronic kidney disease, and is characterized by intractable systemic itching, often without any other obvious cutaneous symptoms (63, 64). IL-31 may play a role in the development and maintenance of uremic pruritus in patients receiving hemodialysis (65). In a study of 178 hemodialysis patients, significantly higher levels of IL-31 were recorded in patients with pruritic symptoms compared with those without (*p* = 0.04) (66).

#### Cholestatic Pruritus

The development of cholestatic pruritus, which is found in patients with primary biliary cirrhosis, primary sclerosing cholangitis, and intrahepatic cholestasis of pregnancy (ICP) (67, 68), may also be linked to an elevation in levels of IL-31. In a small-scale study, median levels of serum IL-31 were found to be significantly higher in 13 pregnant women with ICP than in the control group of 26 pregnant women without ICP (*p* = 0.004); in addition, levels of IL-31 showed a direct correlation with liver transaminase levels (68). Of note, the IL-31 pathway has also been reported to be involved in the pathogenesis of hepatitis B virus-related liver cirrhosis (69), potentially suggesting a wider role for IL-31 in hepatic health.

#### Bullous Pemphigoid and Chronic Urticaria

In autoimmune skin diseases, such as bullous pemphigoid and chronic urticaria, IL-31 may be involved in both pruritus and immunomodulation, potentially underpinning the IgE-associated pathophysiology involved in these diseases (35). Skin (70) and serum (71) samples from patients with chronic urticaria have been shown to have high levels of IL-31, and following stimulation, basophils from normal skin also demonstrated increased IL-31 expression, resulting in the release of pro-inflammatory cytokines and the induction of chemotaxis (70). In bullous pemphigoid, it is eosinophils that appear to be the major source of IL-31 (72, 73). Another, related, condition in which IL**-**31 has been implicated is pemphigus herpetiformis (dermatitis herpetiformis) (74, 75).

#### Allergic Contact Dermatitis (ACD)

Around 20% of adults worldwide are affected by ACD, either as a result of sensitivity to everyday products (particularly fragrances) or via exposure to allergens within their work environment (76). A role for IL-31 in ACD has been postulated, with elevated serum levels of IL-31 found in ACD patients compared with controls (77). However, the precise details remain to be clarified, as a preclinical study has suggested that IL-31 may be associated only with pruritus, but not inflammation in contact hypersensitivity (78).

#### Dermatomyositis

Dermatomyositis is a chronic, inflammatory, autoimmune disease characterized by cutaneous involvement (79). Although symptoms can be heterogeneous, pruritus is a common manifestation (80). A recent analysis indicated that gene and protein expression of IL-31 and IL-31RA was increased in dermatomyositis lesions compared with non-lesional skin and normal control skin (81).

#### Chronic Pruritus of Unknown Origin (CPUO)

CPUO is the nomenclature used to describe the presentation of chronic itch which has no distinct etiology (82). The pathophysiology of CPUO remains unclear (10), but patients with CPUO have been shown to have elevated serum levels of IL-31 compared with healthy subjects (7). In regression analysis the presence of CPUO was found to be independently and significantly associated with serum Il-31 levels (*p* < 0.001) (7).

#### Other Dermatologic Conditions

Other dermatologic conditions in which IL**-**31/IL-31R have been implicated include lichen planus (83), cutaneous (lichen) amyloidosis (84–86), statis dermatitis (87), scleroderma (88), and the itch associated with wound healing (89). However, detailed data are lacking, and further studies are necessary to fully determine the role of IL-31 in the pathophysiology and symptomatology of each of these conditions.

### Non-pruritic Diseases

#### Allergic Asthma

Expression of IL-31RA is tightly regulated within the various cells found in the lung (90), and *in vitro* studies have suggested IL-31-stimulation of bronchial cells results in the production of proinflammatory cytokines, growth factors, and chemokines, which could contribute to the inflammation, tissue damage and pulmonary remodeling observed in asthma (91). Studies have shown that levels of IL-31 mRNA and protein are elevated in patients with allergic asthma (92), and expression of both IL-31 and IL-31R are increased in the bronchial tissue of patients with severe asthma (93). Serum IL-31 levels were also found to positively correlate with asthma severity and IgE levels (93).

#### Inflammatory Bowel Disease (IBD)

Analysis of colonic biopsies from patients with Crohn's disease and ulcerative colitis found that IL-31, IL-31RA, and OSMR mRNA expression was increased in inflamed lesions compared with non-inflamed lesions (94). There has been a great deal of recent interest in the possibility of targeting OSM in IBD (95), but IL-31 does not currently appear to be a drug target for this condition.

#### Osteoporosis

Numerous inflammatory cytokines have been demonstrated to be involved in bone remodeling, and there are both *in vitro* and clinical observations which suggest a role for IL-31 in the development of osteoporosis (96). In a study in postmenopausal females, patients with osteoporosis exhibited elevated levels of serum IL-31 compared with healthy controls (*p* < 0.049) (97). Notably, higher levels of IL-31 were associated with increased age, suggesting an association between Th2 cytokine overexpression and bone resorption in senile osteoporosis (98).

## Current Clinical Status of Anti-IL-31 Therapies

Since current anti-pruritic treatments are often ineffective (99, 100), affected patients are in urgent need of new therapies (101, 102). As a result, drug development targeting the IL-31 pathway is evolving rapidly. An overview is provided in Table 1.

**Table 1:** Summary of anti-IL-31 therapies in development.

**Agent**	**Target**	**Drug attributes**	**Disease**	**Highest development stage**	**Key outcomes**
BMS-981164	IL-31	Monoclonal antibody, administered SC and IV	Atopic dermatitis	Phase 1	• Unknown (completed 2015 but unpublished)
Lokivetmab	IL-31	Caninized monoclonal antibody, administered SC	Canine atopic dermatitis, canine mastitis	N/A (veterinary use only)	• Reduces pruritus in dogs
Nemolizumab	IL-31RA	Humanized monoclonal antibody, administered SC	Atopic dermatitis	Phase 3 (administered with concomitant TCS/TCI) (103)	• Mean percent change in pruritus VAS from baseline to week 16: nemolizumab−42.8%, placebo−21.4% (difference, −21.5%; *p* < 0.001) • Reduction in EASI score: nemolizumab −45.9%, placebo −33.2% • Score of ≤ 4 on DLQI: nemolizumab 40%, placebo 22% • Score of ≤ 7 on ISI: nemolizumab 55%, placebo 21% • Rates of adverse events were similar between treatment groups
			Prurigo nodularis	Phase 2 (104)	• Percent change in the mean peak pruritus NRS score from baseline to week 4: nemolizumab−53.0%, placebo −20.2% (difference −32.8%; *p* < 0.001) • Similar trends observed for secondary outcomes • The overall tolerability profiles were comparable between treatment groups
Vixarelimab	OSMR	Monoclonal antibody, administered SC	Prurigo nodularis	Phase 2a (105)	• LSM change in weekly average WI-NRS from baseline at Week 8: vixarelimab−50.6%, placebo−29.4% (difference 21.1%; *p* = 0.035) • No dose-limiting AEs, no serious AEs, no atopic dermatitis flares
			Chronic pruritic diseases^a^	Phase 2 (105)	• Plaque psoriasis: LSM change in WI-NRS from baseline to Week 8: vixarelimab −66.5%, placebo −29.0% (*p* = 0.012). • Chronic idiopathic pruritus: LSM change in WI-NRS from baseline to Week 8: vixarelimab−52.4%, placebo −48.8% (*p* = 0.813). • No formal statistical analysis in lichen simplex chronicus, chronic idiopathic urticaria, and lichen planus due to small patient numbers (<5 per group) • No dose-limiting AEs

a *Including plaque psoriasis, chronic idiopathic pruritus, lichen simplex chronicus, chronic idiopathic urticaria, or lichen planus*.

*AE, adverse event; DLQI, dermatology life quality index; EASI, eczema area and severity index; IL-31, interleukin-31; IL-31RA, interleukin 31 receptor A; ISI, insomnia severity index; IV, intravenously; LSM, least squares mean; N/A, not applicable; NRS, numeric rating scale; OSMR, oncostatin M receptor; SC, subcutaneously; TCS/TCI, topical corticosteroids/topical calcineurin inhibitors; VAS, visual analog scale; WI-NRS, worst-itch numeric rating scale*.

### Anti-IL-31

Two agents targeting IL-31 have been developed thus far, one intended for clinical use and one for veterinary use.

#### BMS-981164

BMS-981164 was an anti-IL-31 monoclonal antibody targeting circulating IL-31 being developed by Bristol-Myers Squibb. A two-part, phase I, single-dose, dose-escalation study was conducted between 2012 and 2015 to explore the safety and pharmacokinetic profile of BMS-981164 (NCT01614756). The study design was randomized, double-blind, placebo-controlled, and the drug was administered as both SC and IV formulations (0.01 to 3 mg/kg) to healthy volunteers (part 1) and adults with atopic dermatitis (part 2). Adult subjects in part 2 were required to have at least moderate atopic dermatitis (assessed by Physician Global Assessment rating of ≥3 on a scale of 0 to 5) and pruritus severity of at least 7 of 10 on a visual analog scale.

To date, no results from this study have been released. As of 2016, BMS-981164 was no longer listed in the development pipeline of Bristol-Myers Squibb, and no new trials have been announced.

#### Lokivetmab

The amino acid sequence of IL-31 has been shown to vary across species, with the human sequence showing >80% homology with isoforms isolated from several species of monkey, but decreased similarities with canine (54%) and murine (30–31%) forms (106, 107). Despite this variation, IL-31 has been linked with the development of pruritus in multiple mammalian species (1, 106, 108).

Lokivetmab (ZTS-00103289) is a caninized IL-31 monoclonal antibody that has demonstrated efficacy in reducing pruritus in dogs, in various conditions including atopic dermatitis (109–111), and mastocytosis (112). Although not suitable for use in humans, data accruing from this agent could inform the future development of other novel anti-IL-31 therapeutic agents.

### Anti-IL-31RA

To date, the only agent targeting IL-31RA is nemolizumab, a subcutaneously-administered humanized monoclonal antibody manufactured by Chugai Pharmaceutical Co., Ltd., and being developed by Maruho Co., Ltd., in Japan and Galderma SA in Europe and the US (106, 113). This has been the most successful strategy to date, with nemolizumab being the only agent targeting the IL-31 pathway to reach phase 3 development. In addition to its effects on pruritus, nemolizumab has also been evaluated for its effectiveness in improving sleep, daily functioning, and quality of life in patients with atopic dermatitis (103).

#### Nemolizumab

In early-phase clinical studies involving adults with moderate-to-severe atopic dermatitis, nemolizumab showed efficacy in reducing both pruritus and also the skin signs of atopic dermatitis (113–116). In the first in-human study, a single administration decreased pruritus, sleep disturbance, and use of topical hydrocortisone, and was well tolerated (113).

In a phase 2, double-blind, placebo-controlled, 12-week trial, adults with moderate-to-severe atopic dermatitis that was inadequately controlled by topical treatments were randomly assigned to receive subcutaneous nemolizumab (at a dose of 0.1, 0.5, or 2.0 mg/kg) or placebo every 4 weeks (Q4W), or nemolizumab 2.0 mg/kg Q8W (115). The primary end point was the percentage improvement from baseline in the score on the pruritus visual analog scale (VAS) at Week 12, and nemolizumab Q4W was shown to significantly improve pruritus at Week 12 (*p* < 0.01 for all doses vs. placebo). Adverse events occurred at similar frequencies with nemolizumab and placebo; however, more nemolizumab-treated patients reported exacerbations in atopic dermatitis and peripheral edema.

In the long-term (52-week) double-blind extension of the phase 2 study (114), the improvements in the pruritus VAS and Eczema Area and Severity Index (EASI) scores from baseline to Week 12 were maintained or increased from Week 12 to Week 64 with nemolizumab treatment. No new or late-onset safety concerns were identified. The mean decrease in Work Productivity and Activity Impairment-Atopic Dermatitis questionnaire score from baseline at Week 12 was greater in nemolizumab-treated patients compared with those receiving placebo for work productivity and ability to perform daily activities (117). Improvements were sustained through Week 64 of the study.

In a phase 2b, randomized, double-blind, placebo-controlled, dose-ranging study of 24 weeks' duration, nemolizumab produced rapid improvements in cutaneous inflammation and pruritus, which were maintained throughout the treatment period, and had an acceptable safety profile (116).

A 16-week, double-blind, phase 3 trial in patients with atopic dermatitis and moderate-to-severe pruritus and an inadequate response to topical agents was recently conducted in Japan (103). Patients were randomly assigned 2:1 to receive subcutaneous nemolizumab 60 mg or placebo Q4W plus concomitant topical corticosteroids/topical calcineurin inhibitors (TCS/TCI). The primary end point (mean percent change in pruritus VAS from baseline to week 16) was reduced by −42.8% in the nemolizumab group and −21.4% in the placebo group (difference, −21.5%; *p* < 0.001). Secondary efficacy data indicated that nemolizumab provided additional benefits to patients, with a reduction in the EASI score of −45.9% (vs. −33.2% with placebo), more patients with a score of 4 or less on the Dermatology Life Quality Index (40 vs. 22% with placebo), and more patients with a score of 7 or less on the Insomnia Severity Index (55 vs. 21% with placebo). Rates of adverse events were similar between treatment groups. Cytokine abnormalities (increased thymus and activation-regulated chemokine level) occurred in the nemolizumab group after treatment; however, there was no association with the EASI score.

Nemolizumab has also demonstrated efficacy in the reduction of pruritus in patients with moderate-to-severe prurigo nodularis and severe pruritus (104). A 12-week, randomized, double-blind, placebo-controlled, phase 2 trial of nemolizumab 0.5 mg/kg administered at baseline, week 4, and week 8 was conducted. The primary outcome (percent change from baseline in the mean peak score for pruritus on the numeric rating scale at week 4) was reduced by −53.0% with nemolizumab vs.−20.2% in the placebo group (difference −32.8%; *p* < 0.001). Secondary outcomes followed the same trend as the primary outcome. Nemolizumab was associated with gastrointestinal symptoms (abdominal pain and diarrhea; 21% vs. placebo 14%) and musculoskeletal symptoms (18% vs. placebo 14%); however, the overall tolerability profile was comparable with that of placebo and only 2 patients in each group discontinued treatment due to adverse events.

### Anti-OSMR

Although modulation of the oncostatin M receptor is potentially of clinical interest, to date, only one drug which directly targets OSMR has been evaluated in clinical trials, and few published details are available.

#### Vixarelimab (KPL-716)

Vixarelimab is a monoclonal antibody being developed for the treatment pf prurigo nodularis by Kiniksa Pharmaceuticals Corp; it simultaneously targets both the OSMRβ, which mediates signaling of IL-31, and the oncostatin M pathways (105). It is subcutaneously administered as a loading dose of 720 mg followed by weekly injections of 360 mg.

In a randomized, double-blind, placebo-controlled phase 2a clinical trial of vixarelimab in 49 patients with prurigo nodularis, vixarelimab met its primary efficacy endpoint of the reduction in weekly average Worst-Itch Numeric Rating Scale (WI-NRS) from baseline at Week 8. At Week 8, the least squares mean change from baseline in weekly average WI-NRS was−50.6% in the vixarelimab treatment group compared with −29.4% in the placebo group (mean difference 21.1%; *p* = 0.035).

In an exploratory phase 2 study, the efficacy of vixarelimab was evaluated in patients with other chronic pruritic diseases, including plaque psoriasis, chronic idiopathic pruritus, lichen simplex chronicus, chronic idiopathic urticaria, or lichen planus. In the plaque psoriasis cohort, the least squares mean change in WI-NRS from baseline to Week 8 was −66.5% in the vixarelimab group and −29.0% in the placebo group (*p* = 0.012). In the chronic idiopathic pruritus cohort, the changes were−52.4% with vixarelimab and−48.8% with placebo (*p* = 0.813). Due to small numbers of patients (<5 per group) with lichen simplex chronicus, chronic idiopathic urticaria, and lichen planus, no formal statistical analysis was performed, but the data were reported to show an encouraging effect of treatment.

In both studies, vixarelimab was said to be well-tolerated, with no dose-limiting adverse events; moreover, no serious adverse events or atopic dermatitis flares were noted in the phase 2a study. However, to date, the only available information has been provided on the manufacturer website (105); no publications are available and the data have not been peer-reviewed. In addition, no phase 3 studies have yet been planned.

## Discussion

Pruritus affects patients of all ages, races, and sex, and can have an extremely negative impact on their quality of life (118). Given that many patients are refractory to available treatments (99, 100), it is important to develop new drugs to improve clinical and social outcomes.

Based on its roles in the development of itch, skin deficits, and inflammation (4, 12–14, 119), targeting the IL-31 pathway is a logical step in the development of new pharmacologic agents against pruritus. To date, however, only the anti-IL-31RA antibody nemolizumab has progressed to late-stage clinical trials. Based on the positive results of the recent phase 3 clinical trial of nemolizumab plus concomitant topical agents to treat patients with atopic dermatitis and moderate-to-severe pruritus (103), and the efficacy benefits shown in reducing pruritus in patients with moderate-to-severe prurigo nodularis (104), this drug appears to hold new hope for patients whose treatment options are currently limited. Moreover, the administration of nemolizumab alongside topical agents closely mirrors the likely real-world clinical situation, in which many patients with atopic dermatitis are already treating their conditions, making the addition of nemolizumab into an ongoing treatment regimen relatively straightforward.

For several of the other diseases discussed herein, the underlying role of IL-31 in their pathophysiology remains unclear or unproven. The notable heterogeneity in IL-31 levels, ranging from >1,000 pg/mL among patients with atopic dermatitis (40) to <200 pg/mL in other diseases (7, 65, 93, 97), requires additional explanation, and further research is clearly warranted.

In addition to IL-31, there has been an explosion of recent interest in novel antipruritic drugs targeting other pathways thought to play role in the development or maintenance of itch, including opioid receptor agonists and antagonists, antibodies against various IL family members, and Janus kinase inhibitors (15, 120, 121). It remains to be seen how many of these drugs will successfully demonstrate long-term effectiveness in controlling the signs and symptoms of pruritus, but it seems likely that the current management algorithms for pruritus will undergo extensive modification over the next few years.

In conclusion, IL-31 plays an important role in several inflammatory skin diseases, and treatment targeting IL-31 is expected to contribute meaningfully to the clinical management of a wide range of diseases.

## Author Contributions

KK and HI were involved in writing and critically reviewing the manuscript and both authors provided final approval of the manuscript for submission and agree to be accountable for the content.

## Conflict of Interest

KK has received grants from Japan Tobacco Inc., Kyowa Kirin, LEO Pharma, Maruho, Mitsubishi Tanabe Pharma, ONO PHARMACEUTICAL, POLA PHARMA, TAIHO PHARMA, Torii Pharmaceutical, and The Procter & Gamble Company, and has received personal fees from Maruho. HI has received grants from Maruho.
